# Identification of Robust Protein Associations With COVID-19 Disease Based on Five Clinical Studies

**DOI:** 10.3389/fimmu.2021.781100

**Published:** 2022-01-25

**Authors:** Karsten Suhre, Hina Sarwath, Rudolf Engelke, Muhammad Umar Sohail, Soo Jung Cho, William Whalen, Sergio Alvarez-Mulett, Jan Krumsiek, Augustine M. K. Choi, Frank Schmidt

**Affiliations:** ^1^ Bioinformatics Core, Weill Cornell Medicine-Qatar, Doha, Qatar; ^2^ Department of Physiology and Biophysics, Weill Cornell Medicine, New York, NY, United States; ^3^ Proteomics Core, Weill Cornell Medicine-Qatar, Doha, Qatar; ^4^ Department of Medicine, Division of Pulmonary and Critical Care Medicine, New York-Presbyterian Hospital-Weill Cornell Medical Center, Weill Cornell Medicine, New York, NY, United States; ^5^ Englander Institute for Precision Medicine, Weill Cornell Medicine, New York, NY, United States

**Keywords:** COVID-19, Olink proteomics, inflammation and cardiovascular markers, cytokine storm syndrome, CCL16, CXCL10, IL6, IL18

## Abstract

Multiple studies have investigated the role of blood circulating proteins in COVID-19 disease using the Olink affinity proteomics platform. However, study inclusion criteria and sample collection conditions varied between studies, leading to sometimes incongruent associations. To identify the most robust protein markers of the disease and the underlying pathways that are relevant under all conditions, it is essential to identify proteins that replicate most widely. Here we combined the Olink proteomics profiles of two newly recruited COVID-19 studies (N=68 and N=98) with those of three previously published COVID-19 studies (N=383, N=83, N=57). For these studies, three Olink panels (Inflammation and Cardiovascular II & III) with 253 unique proteins were compared. Case/control analysis revealed thirteen proteins (CCL16, CCL7, CXCL10, CCL8, LGALS9, CXCL11, IL1RN, CCL2, CD274, IL6, IL18, MERTK, IFNγ, and IL18R1) that were differentially expressed in COVID-19 patients in all five studies. Except CCL16, which was higher in controls, all proteins were overexpressed in COVID-19 patients. Pathway analysis revealed concordant trends across all studies with pathways related to cytokine-cytokine interaction, IL18 signaling, fluid shear stress and rheumatoid arthritis. Our results reaffirm previous findings related to a COVID-19 cytokine storm syndrome. Cross-study robustness of COVID-19 specific protein expression profiles support the utility of affinity proteomics as a tool and for the identification of potential therapeutic targets.

## Introduction

The recent COVID-19 pandemic has brought global socio-economic activity to a halt and devastated major health-care systems all over the world. In most individuals, COVID-19 manifests as an asymptomatic or a mild respiratory tract infection (1). However, the disease may exacerbate to severe pneumonia or acute respiratory distress syndrome (ARDS) in some individuals, especially in those with high age, obesity, diabetes, and other underlying comorbidities. A cytokine storm syndrome has been associated with a large portion of the mortality in patients hospitalized with COVID-19 (2).

The number of COVID-19 positive cases is rising every day and can be tracked in real time, e.g., on the Johns Hopkins University website (3). Until the end of March 2021, the United States, Brazil, and most European countries had been the epicenters of the disease, with the highest morbidity and mortality rates (4). However, in the middle of April, the incidence rate in India skyrocketed due to the emergence of novel mutations in the virus, which questioned the effectiveness of existing vaccines and resulted in substantially higher mortality rates (5). WHO has recently announced a fourth wave of virus outbreaks, this time involving the delta version of the virus, which pushed some countries that had lifted and then re-imposed restrictions. The virus shares a close genetic resemblance with SARS-CoV and Middle East Respiratory Syndrome (MERS), the infectious agent that caused the 2002 SARS and 2012 MERS epidemics, respectively (6).

The paucity of accurate molecular markers makes it difficult to track disease progression, and currently disease prognosis is mainly dependent on clinical manifestations. Historically, it is thought that an immune exacerbation contributed to the high fatality of the 1918–1919 influenza pandemic (7). Likewise, prior coronavirus pandemics (SARS and MERS) also reported severe hypercytokinemia and lymphocytopenia as the specific disease severity markers (8, 9). Fajgenbaum and June (10) suggested that pathogenesis of COVID-19 disease is strongly associated with acute hyperinflammatory reaction characterized by hypercytokinemia, coined the term ‘cytokine storm syndrome’ (CSS).

CSS is a natural defense response of overactive immune cells such as B cells, T cells, dendritic cells, macrophages, and natural killer cells, which release tremendous amounts of inflammatory cytokines. Consequently, more immune cells are activated, creating a positive feedback loop (11). This form of hyper immune activation is associated with acute disease progression and poor therapeutic response. Therefore, it is vitally essential to identify specific immunomodulatory and tissue-associated protein markers related with COVID-19 for providing insights into beneficial and detrimental host responses. Exploring infection and immune inflammatory pathways responsible for disease pathogenesis and critical outcomes. Specifically, comparing key regulatory pathways in COVID-19 patients to the same degree regardless of patient ethnicity, blood matrix, disease phase, or study design.

According to a recent NIH/FDA COVID-19 conference, the core aim of COVID-19 research should be to find out how and why SARS-CoV-2 induces heterogeneity in disease severity and immunopathology across infected populations (12). To date, several studies have explored COVID-19 immunodynamics in small and relatively diverse population samples, addressing larger numbers of immune parameters (13–16). However, there is a lack of continuity in the findings across studies. To develop new diagnostic, prognostic, and therapeutic methods, recent research is crucial in gaining a better understanding of the cellular pathways underlying SARS-CoV-2-induced immune-inflammatory interactions. Particularly, cytokine profiling using high throughput proteomics tools is deemed essential in COVID-19 management and therapeutic quest.

We used the Olink Inflammation and Cardiovascular panels II & III containing 266 relevant unique proteins to describe the host proteomic responses to COVID-19 in two population cohorts of acutely ill patients reporting at New York Presbyterian Hospital/Weill Cornell Medical Center, General Internal Medicine ward (GIM) and Intensive Care Unit (ICU). These data were compared with that from three previously published studies (Massachusetts General Hospital (MGH), Boston (15), and Imperial College Healthcare NHS Trust (IMP and REP) (17) to identify proteins that are robustly associated with immune cell activation, cytokine syndrome.

## Results

### Cohort Design and Demographics of the Five Cohorts

The ICU cohort is composed of 43 patients who met criteria for ARDS as defined by the Berlin definition (18) and tested positive for SARS-CoV-2 by RT-PCR, while 25 nonsepsis ICU patients served as controls. The GIM cohort was comprised of 48 patients with confirmed SARS-CoV-2 RT-PCR, and 50 non-COVID-19 controls with negative RT-PCR results who were hospitalized at the New York-Presbyterian Hospital/Weill Cornell Medical Center between March and April 2020. A detailed description of the demographic characteristics of the cohorts can be found in **Table 1**. Details about the MGH, IMP, and REP studies can be found at (15, 17). In brief, MGH study enrolled 306 confirmed COVID-19 patients who were presented to emergency department of Massachusetts General Hospital. All the patients were classified into five acuity levels based upon disease severity and clinical outcomes (19). The IMP and REP studies included 55 and 46 (respectively) COVID-19 positive end-stage kidney disease (ESKD) patients and 51 and 11 non-infected hemodialysis patients as ESKD controls, which matched the age, sex, and ethnicity of the COVID-19 cases. Patients for IMP and REP studies were recruited at Imperial College Renal and Transplant Centre and its satellite dialysis units.

**Table 1:** Demographic and clinical characteristics of the five COVID-19 study cohorts.

Study Samples	COVID-19 Patient Metadata									
#Case vs. Control	Age median year (IQR)	Gender[%]	Co-morbidities [%]	Smoking[%]	BMI[IQR]	COVID-19 Severity [%]	Ethnicity[%]	Olink panels	Sampling matrix	Reference
ICU 43 vs. 25	58.7(17.7)	M=86 F=14	Heart diseases=12 Diabetes=16 Hypertension=40 Acute kidney injury=61	4.6	27.4(6.5)	Severe ARDS=100	White=35 Black=7 Asia=9 Other=49	Infl., CVD II & III	Plasma	NA
GIM 48 vs. 50	66.1(25.2)	M=60 F=40	Heart diseases=15 Cancer=15 Diabetes=25 Hypertension=44 ESRD=13 Chronic kidney disease=4 Acute kidney injury=10	0.0	27.1(7.8)	Mild/Moderate=63 Severe=37	White=50 Black=13 Asia=4 Other=33	Infl., CVD II & III	Serum	No reference available
^*^IMP 33 vs. 50	72.2(14.8)	M=71 F=29	Diabetes=62 ESKD=100	1.8	NA	Mild/Moderate=51 Severe=49	White=29 Black=15 South Asia=33 Asia=7 Others=16	Infl., Immune Response CVD II & III	Plasma	(17)
^*^REP 52 vs. 11	64.3(12.7)	M=70 F=30	Diabetes=63 ESKD=100	4.3	NA	Mild/Moderate=29 Severe=71	White=24 Black=17 South Asia=26 Asia=15 Others=17	Infl., Immune Response CVD II & III	Serum	(17)
^*^MGH 305 vs. 78	58.0(30.)	M=53 F=47	Heart diseases=16 Diabetes=36 Hypertension=48 Hyperlipidemia=22 Chronic lung disease=22 Kidney disease=13 Immunocompromised =8	3.6	29(8.0)	Mild/Moderate=69 Severe=31	Hispanic=54 Black=10 Not described=36	Olink^®^ Explore 1536	Plasma	(15)

^*^These are the numbers used in the current analysis after removal of additional samples that have been measured from the same patients on successive time-points. NA, Not available.

### Olink Proteins Intensity Distribution

**Figure 1:**
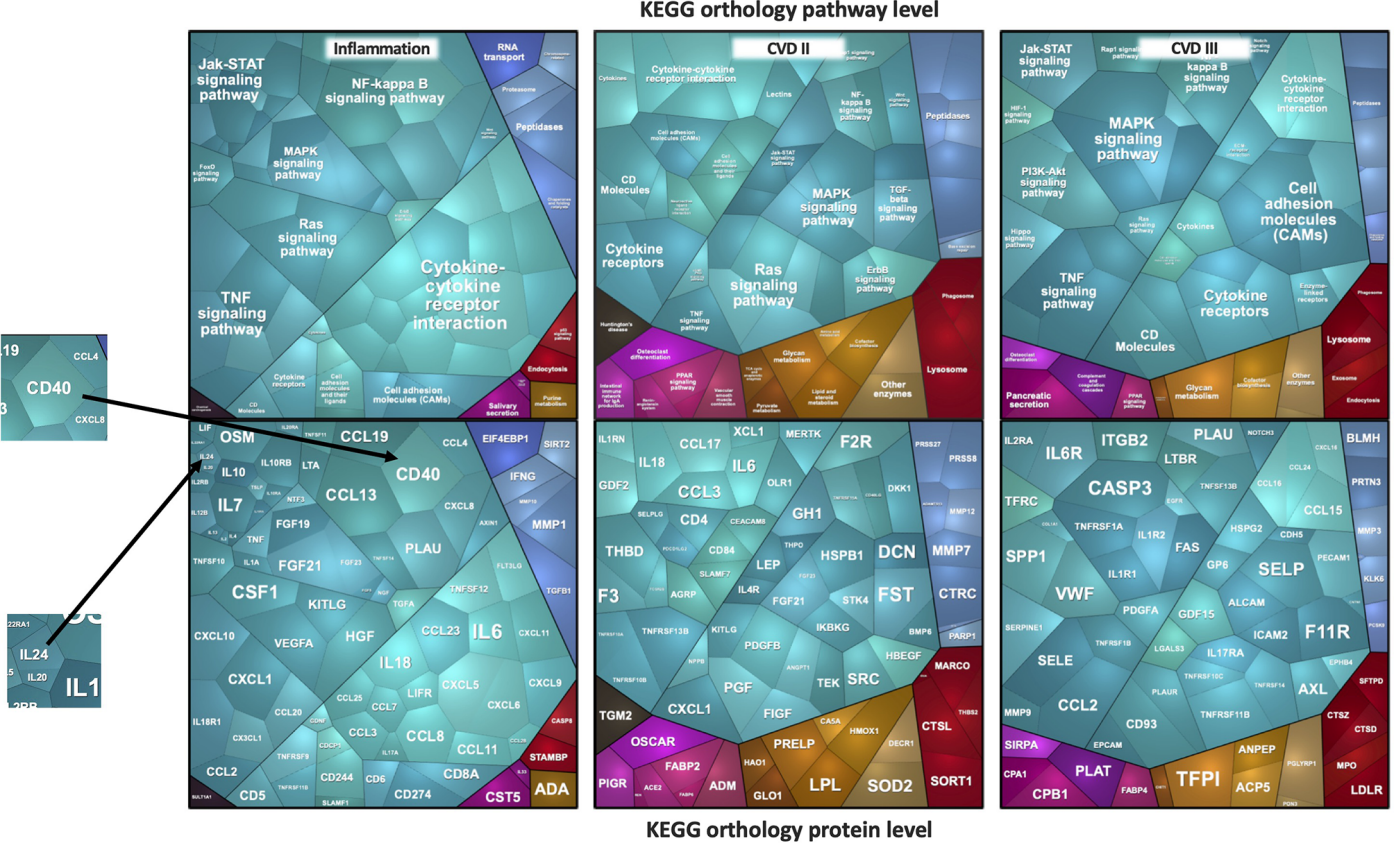
Voronoi treemaps of proteins measured by the Olink platform. The median log_2_ intensities of proteins expressed as cell sizes in ICU plasma samples are depicted. The sizes of the polygons reflect protein intensity levels. In the top figures, panel-specific pathways are shown and grouped by identical color schemes if they belong to the same pathway. In the bottom figures, the corresponding protein names are shown.

For inter-cohort analysis, we used the intersection of all proteins from the Olink panels that were measured in all five cohorts, namely Cardiovascular II & III, and Inflammation panels, which comprised 92 proteins each. A total of 276 proteins were measured in all five studies. Ten proteins were present as duplicates in cardiovascular and inflammation panels, leaving 266 unique proteins in total. Blood circulating levels of these proteins were recorded as normalized protein expression (NPX) values. Samples that had NPX values below the protein-specific limit of detection (LOD) in more than 50% of samples were excluded, leaving 253 proteins for analysis. To get an impression of the protein intensity distribution, the medians of logarithmic intensities from the ICU cohort were plotted exemplary on a Voronoi treemap to get a global view of the protein intensities detected with Olink (**Figure 1**). The Voronoi treemaps use protein annotation information from KEGG BRITE to present individual protein tiles linked to functional pathways (20). The advantage of KEGG BRITE is that each protein is assigned to only one pathway, thus avoiding redundancies.

The Voronoi treemaps displayed in **Figure 1** represent individual proteins that correspond to specific functional pathways. Related proteins/pathway connections are presented in identical colors, and the sizes of the polygons reflect protein intensity levels. To give an example, in the ICU study, the most densely covered pathways are cytokine-cytokine interaction, NF-kappa B-, TNF-alpha-, JAK- or MAPK-, and RAS-signaling pathways. Interleukins for example, mainly part of the JAK-signaling pathway or partly the cytokine-cytokine interaction, showed remarkable lower intensities in the plasma samples. Proteins belonging NF-kappa B-, TNF-alpha- or MAPK-signaling, on the other hand, were found in higher abundances. One of the most abundant proteins is CD40 from the NF-κB signaling pathway, one with the lowest abundance is IL24. Similarly, FST, CXCL1, CCL2, and CASP3 were the most abundantly expressed proteins from cardiovascular panels that belonged to TGF-beta-, TNF-, and MAPK-signaling pathways.

### Differential Analysis of Five Olink Studies Based on 253 Common Proteins

**Figure 2:**
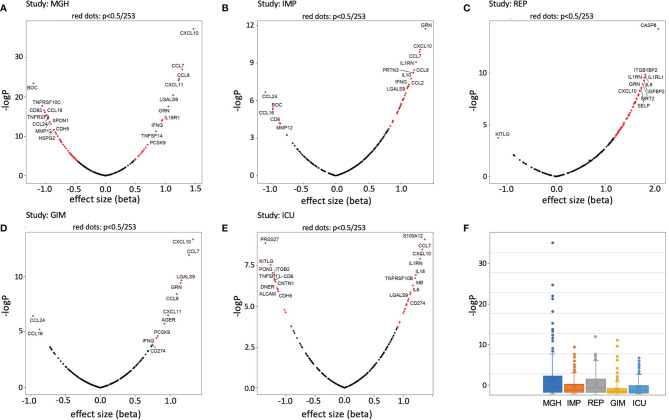
Volcano plots and boxplots of differentially expressed proteins for MGH **(A)**, IMP **(B)**, REP **(C)**, GIM NY **(D)**, and ICU NY **(E)** studies. Proteins showing significant differences between case and control are shown in red. Change x-axis indicating control high on the left and COVID-19 high on the right. The top 10 proteins with the highest effect sizes and high p-values have been highlighted. In addition, p-value (-logP) boxplots of each study are shown in **(F)**.

In a first approach, we calculated the difference of the means and the corresponding p-values (unrelated T-test) to identify proteins that were significantly altered in a case vs. control design. To visualize the associations, volcano plots were created for the 253 proteins. For this analysis, the data was scaled to a mean of zero and a standard deviation (s.d.) of one, as previously done for other studies (see Methods). It can be clearly seen that the effect sizes in IMP (**Figure 2B**) and REP (**Figure 2C**) tend to show significantly more upregulation in COVID-19 than, for example, in MGH, GIM, and ICU (**Figures 2A, D**, **E**), where a more even distribution is observed. The -log p-values of the Olink Explorer platform used in MGH (**Figure 2F**) were significantly higher than the -log p-values of the Olink Panel platform used in all other studies, with somehow similar effect sizes (beta).

**Figure 3:**
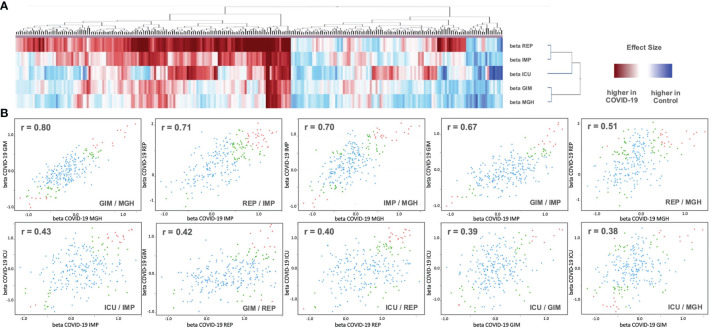
**(A)** Analysis of 2D cluster pattern of all 253 beta values from the five studies is displayed. Coloring is based on beta values, with a higher abundancy in control in blue and a higher abundancy in COVID-19 in red. **(B)** Shown are 10 intergroup scatter plots of effect estimates from case vs. control ratios. Red indicates proteins that were Bonferroni significant at discovery level in one study (p < 0.05/2/Nprotein) and at replication level in the other (p < 0.05/Ndiscovery), where Nprotein ist the number of proteins tested and Ndiscovery the number of proteins reaching discovery significance in the first study. Green are proteins that reach nominal significance (p<0.05) in both studies. All other proteins are in blue (see **Supplementary Data 1** for full summary statistics).

To find general similarities/dissimilarities between the studies, we applied 2D clustering based on the differences in the means (beta values) (**Figure 3**). Two main clusters with small distances (high similarity) were observed for REP and IMP, and GIM and MGH. ICU tended to be more related to REP and IMP. These three studies generally have more changes between COVID-19 and control compared with GIM or MGH. In addition, pairwise correlation of effect sizes between studies is presented in scatterplots. Two studies, GIM vs. MGH, show the highest correlation between effect estimates (r = 0.80), suggesting that they are more similar than ICU, IMP, and REP. The second and third highest correlation values were observed for REP vs. IMP (r = 0.71) and IMP vs. MGH (r = 0.70). The ICU study was slightly outside and showed only low levels of agreement (r < 0.50) compared with the other studies, as shown in **Figure 3** below. Further, the beta values of the REP study showed the strongest differences compared to the other studies, with a tendency toward upregulation. In part, this is also true for values that are downregulated in all other studies, which could be due to an artificial or technical effect.

**Figure 4:**
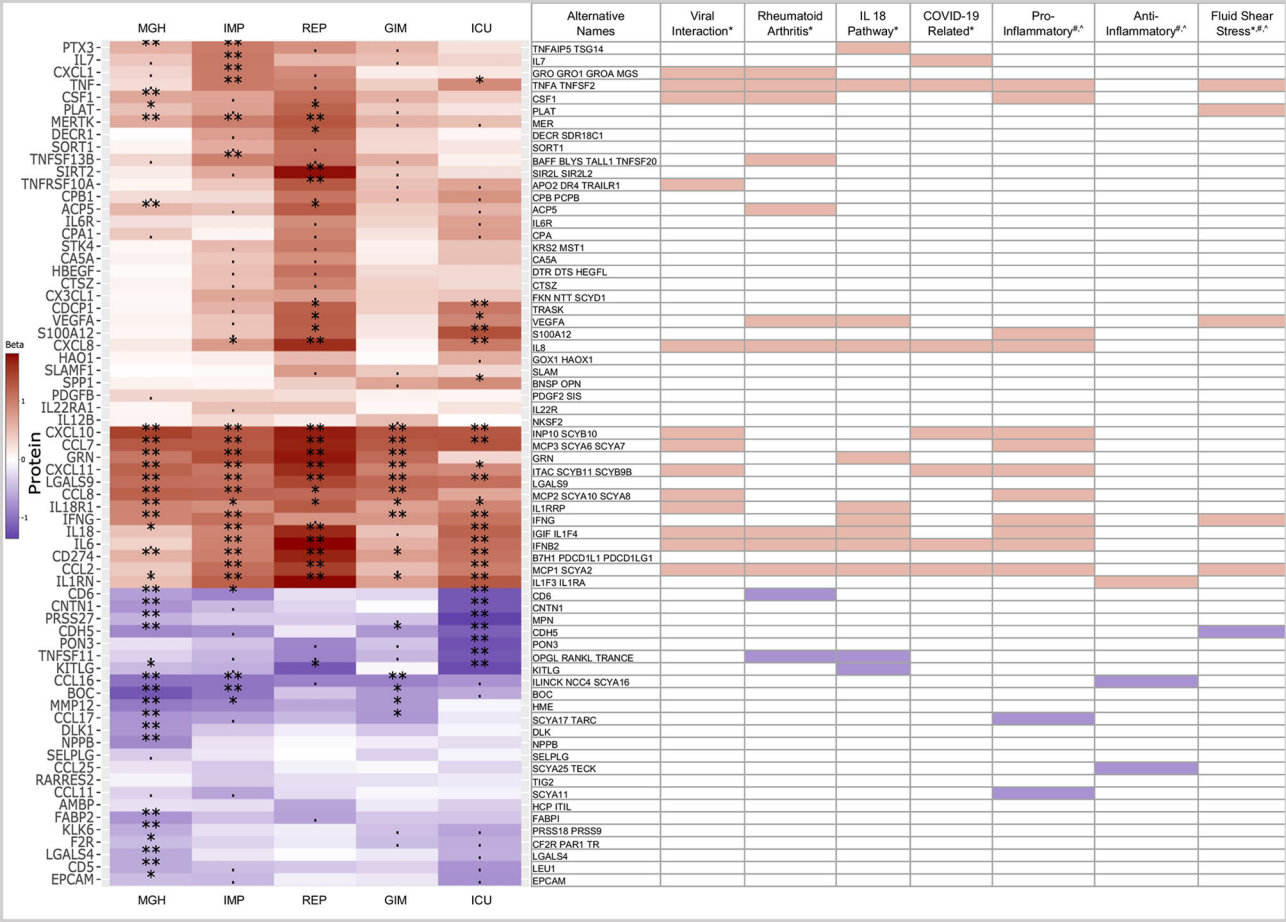
Heatmap of 68 proteins differentially expressed across all five studies. Limited to proteins with similar trends (red: higher in case, blue: higher in controls), significance levels are indicated by ‘**‘ (p<0.05/253/5 = 4x10^-5^, Bonferroni level), ‘*‘ (p<0.05/116 = 0.00043, replication level, as there were 116 Bonferroni significant associations), “. “ (p<0.05, nominal significance). Selected assigned pathways/groups are shown. Pathway information was retrieved form g:Profiler (*), Uniprot (#) or literature analyses (^). The g:Profiler analysis included only pathways from KEGG, Reactome and Wikipathway.

We were particularly interested in proteins that were consistently associated with COVID-19 across all five studies. We therefore required at least one protein in a study that could be at a Bonferroni significance level of less than 0.05, corrected for testing 253 proteins for association in five studies (p < 0.05/5/253 = 3.95x10^-5^), with the same direction of association in the remaining four studies (**Figure 4**). From this dataset, we further extracted those proteins that were also differentially expressed (p<0.05) in the same direction in all studies.

In the heatmap in **Figure 4**, 68 of 253 proteins show consistent expression patterns across all five studies. In the COVID-19 cases, 44 proteins showed higher abundances, while 24 proteins showed lower abundances. Below, we further investigated to which pathways or groups the alternating proteins were assigned.

Many of the upregulated proteins could be assigned to pathways or groups associated with viral infection, such as general viral protein interaction with cytokine and cytokine receptors from the KEGG pathway (04061) or COVID-19 adverse outcome (WP4891) and innate immunity evasion (WP5039) pathways from Wikipathway. Thirteen proteins were part of the viral interaction pathway, with the majority of proteins assigned to the cytokine family. In addition to the virus-specific pathways, 12 proteins were also assigned to the rheumatoid arthritis pathway, 12 to the IL18 (WP 4754) or 6 to the fluid shear stress pathways. Many of these proteins are part of the pro-inflammatory and not of the anti-inflammatory system. This is an intriguing finding in COVID-19, considering pro- and anti-inflammatory pathways are engaged at the same time in most other disease states (21).

Thirteen proteins were part of the viral interaction pathway, with most proteins assigned to the cytokine family such as CCL17, CCL16, CCL2, CCL8, CXCL11, CCL7, CXCL10, CXCL1, and CXCL8. The cytokines are complemented by infection and inflammation related proteins such as IL6 or IL18 and TNF, the key players in infectious disease states with both beneficial and harmful, pleiotropic activities (22).

Twelve proteins were assigned to the rheumatoid arthritis, an autoimmune condition pathway, caused by the immune system attacking healthy body tissue. Seven proteins were shared with the viral interaction pathway and five were unique compared to viral interaction such as TNFSF13B, TNFSF11, CD6, VEGFA, and ACP5.

Twelve proteins were assigned to the IL18 pathway, a host defense pathway following bacterial or viral infection that is activated *via* an interplay between the inflammasome and IL1-beta and IL18 (23). Recently, an *in vitro* study used humanized anti-IL1R7 antibody to suppress IL18-mediated inflammatory pathways, including NFκB activation and IFNγ, IL6, and TNFα production (24). In comparison to other pathways or groups, three interaction proteins, KITLG, GRN, and PTX3, are rather unique that play significant role in several physiological processes. The KIT ligand is binding to SCF and involved in several pathways such proliferation, hematopoiesis or stem cell maintenance (25). GRN is involved in inflammation and wound healing or proliferation and the acidification of lysosomes, a key response to external pathogen stimulus. PTX3 regulates the innate resistance to pathogens and is involved in the inflammatory reactions (26).

Furthermore, three out of the six proteins namely CDH5, PLAT, and VEGFA are unique to the fluid shear stress and atherosclerosis pathway, a pathway which is involved in the tangential stress due to the friction of the flowing blood on the endothelial surface of the arterial wall (27).

### Most Strictly Similar Protein Alterations in All Five Studies

**Figure 5:**
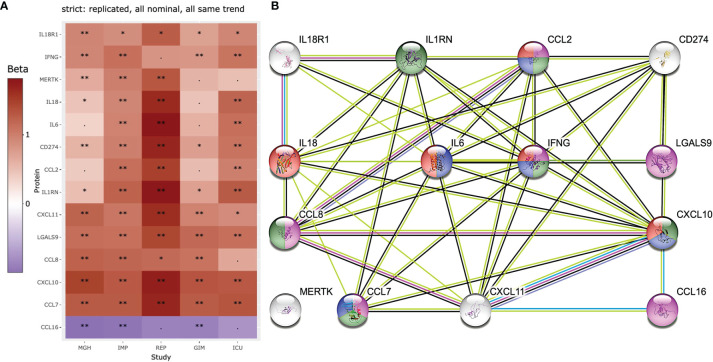
Heatmap and STRING analysis of the 14 most stringent and replicated proteins in all 5 studies are shown. **(A)** 13 of the 253 proteins showed similar significantly increased and decreased levels in COVID-19 patients. **(B)** proteins were further used for STRING protein interaction analysis and compounds of interest are highlighted. The proteins highlighted in purple are known to be involved in general IFN gamma cell responses to external stimuli by pathogens (e.g., viruses or bacteria: GO:0071346). Proteins highlighted in green (e.g., CCL2=MCP-1, CCL7=MCP-3, CCL8=MCP-2, CXCL10=INP10, are proteins known to be elevated in blood after infection for example with *M. tuberculosis* (28). Proteins in red are associated with influenza A infection (hsa05164) and blue with the IL17 signaling (hsa04657). Significance levels are indicated by ‘**‘ (p<0.05/253/5 = 4x10-5, Bonferroni level), ‘*‘ (p<0.05/116 = 0.00043, replication level, as there were 116 Bonferroni significant associations), “. “ (p<0.05, nominal significance).

One of the pressing questions we aim to answer with this study comparison is which of the regulated proteins are up- or down-regulated to the same extent in COVID-19 patients compared to controls, regardless of patient nationality, blood matrix, COVID-19 inactivation process, or study design. For this reason, only those proteins that showed strict similarities in beta and p-values were further compared, as shown in **Figure 5**.

Three key proteins generally involved in infection and inflammation (IL6 IL18, and IFNG) are the focus of this analysis and are associated with 10 other proteins. Only MERTK, a tyrosine protein kinase, showed no direct association with any other of the 13 proteins. The proteins highlighted in purple are known to be involved in general IFN gamma cell responses to external stimuli by pathogens (e.g., viruses or bacteria: GO:0071346). Proteins highlighted in green (e.g., CCL2=MCP-1, CCL7=MCP-3, CCL8=MCP-2, CXCL10=INP10, are proteins known to be elevated in blood after infection for example with *M. tuberculosis* (28). Proteins in red are associated with influenza A infection (hsa05164) and blue with the IL17 signaling (hsa04657). It is known that IL18 and R1 are part of the IL18 pathway, but this pathway did not appear in the top rankings of STRING using the 14 proteins.

## Discussion

Serum proteomics of COVID-19 patients has been documented in multiple studies, often stratified by disease severity and comorbidities, representing IL6 concentrations as a marker of disease severity and prognosis (17, 29, 30). Many of these studies highlight significant changes in immune-inflammatory pathways, predicting inflammatory cytokine signature for COVID-19 severity and survival (31–33). Although there were some overlaps in findings, particularly in terms of IL6 overexpression and acute phase responses, not all the studies reveal consistent observations, and the candidate proteins in the so-called cytokine storm syndrome differ a lot depending on the population’s demography.

The variations in protein expression in COVID-19 patients between five case-control studies were investigated in this paper. All COVID-19 patients, regardless of disease status (mild, severe, and critical), were grouped together as the COVID-19 case group and compared with control group to see differential expression of proteins (19). Case-control comparisons were used to translate all data into differences between the means and p-values for each protein. All studies measured a joint overlapping set of 253 proteins, which were analyzed here. Only the first data point per patient was used for comparison when times-series data was available for longitudinal studies (IMP, REP, and MGH) (15, 17).

In the ICU study, over thirty-seven proteins were substantially altered, with twenty-three being overexpressed and fourteen being declined. Around twelve proteins were significantly perturbed in the GIM study, with ten being overexpressed and two being inhibited. Similarly, IMP, REP, and MGH studies reported seventy-seven, forty-one, and ninety-three proteins, being altered, respectively. However, comparison among these large-scale studies suggests that the number of consistently quantified proteins is more relevant than the maximum number of proteins being altered, as only consistent detection allows for quantitative comparison between individuals and is suitable for the development of clinical assays. Correlation analysis revealed a core panel of 68 proteins that exhibited same expression pattern across all the studies. Not surprisingly, there was a high degree of similarity in protein expression among the studies. Particularly, MGH and GIM exhibited most similarities (r = 0.80), followed by IMP and REP (r = 0.71), and MGH and IMP (r = 0.70). These cross-continental comparisons are essential for the discovery of novel diagnostic and prognostic markers. Future studies should include similar comparisons from other ethnicities to bolster our results and offer a more universal proteomics profile.

Pietzner et al. (34) categorized proteomics data based upon the proteins’ role in COVID-19 pathology, including disease severity, complications, and therapeutic markers. Many of the cytokines that were overexpressed in our research cohorts, including IL1 and IL6, were identified as COVID-19 related CSS proteins. Strictly replicated proteins in all five studies identified several inflammatory mediators associated with death in ARDS patients, including previously identified markers (IL1RN, IL6, IL18, IL18R1, IFNγ, and CXCL10) as well as several novel markers (CCL2, CCL7, CCL8, CCL20, CXCL11, AREG, IL1RL1, FLT1 and IL24). The cytokine profile in these studies is comparable to that reported in cytokine release syndromes, such as macrophage activation syndrome, which is characterized by increased expression of cytokines (IL6, IL7, and TNF) and inflammatory chemokines (CCL2, CCL7, CXC-10, and CXCL-11) (35). Additionally, these findings point to a possible secondary bacterial infection in the critically ill COVID-19 patients, and may lead to peripheral organ failure (e.g., kidney), as shown by MGH study comparing proteomics and kidney function tests (15).

By leveraging proteomics datasets that shared similar expression pattern, we identified a subset of proteins highly elevated among all the studies, deconvoluting their relative contribution in immune-inflammatory pathways. Several immune pathways were activated in the COVID-19 patients, including viral protein interaction with cytokine and cytokine receptors, and COVID-19 adverse outcome pathway. Particularly, Wikipathway COVID-19 adverse outcome pathway regulates leukocyte activation by involving toll-like receptor signaling and inflammatory cytokines responses. The pathway involves several immune-inflammatory cytokines, including CCL3 and CXCL10 in receptor signaling, IL1B, IL2, IL6, IL7, IL10, TNF, CSF3 in inflammatory responses, and IL2RA and CCL2 in leukocytes activation (36). In our study, many of these cytokines and chemokines, especially CCL2, CXCL10, and IL6, were found to be significantly overexpressed in COVID-19 patients. Even when the five studies were compared, most of these cytokines were shown to be overexpressed, indicating that the COVID-19 adverse outcome pathway was activated in all of them.

In a positive feedback loop, these and other immune pathways activate the cellular and adaptive immune system. Immune-inflammatory studies of viral infections point to several mechanisms that contribute to mediating inflammatory responses. One of these is the interaction of viral proteins with cytokines and cytokine receptors initiated by the chemokine subfamilies CCL and CXL 25. Furthermore, consistently unique cytokine expression patterns of regenerative and growth factors (CCL2, CCL11, IL6, IL12B, and CXCL8) may indicate activation of pulmonary fibrosis signaling pathways after aberrant inflammatory response.

However, none of the groups or pathways described here are exclusive to COVID-19. Many of the proteins highlighted here are already attributed to other external stimuli such as bacteria, yeast, other viruses, or even allergens. We should be cautious in interpreting the common proteins discovered here, as many of them can be activated by key players of human defense such as IFNG, IL6 or TNF, leading to common pleiotropic effects. We further should not use the term COVID-19 biomarker at this point, a misunderstanding that has already led to confusion in the field of bacterial sepsis, allergy, diabetes but also cancer, because the selectivity is not convincing. Overexpression of IL6, for example, as reported in several COVID-19 studies, did not provide an effective clinical signal for COVID-19 treatment in an initial clinical trial (COVACTA) (36). Tocilizumab, an anti-IL6 medication, was tried on COVID-19 patients but was shown to be ineffective in a randomized controlled trial (36). Whereas Tocilizumab enhanced survival and other clinical outcomes in follow-up trials of hospitalized patients (RECOVERY) and ICU patients (REMAP-CAP) (37, 38). IL18, on the other hand, is one of the most important cytokines in macrophage activation syndrome, which has not been thoroughly studied in COVID-19. Satış et al. (39) reported high serum concentrations of IL18 that correlated with other inflammatory markers, which is also consistent with our findings. A candidate COVID-19 therapy is an anti-human IL1R7 antibody that suppresses IL18-mediated inflammatory signaling (24). For these reasons, it is necessary to perform comparative multi-disease studies with e.g., bacterial sepsis, allergy, and diabetes patients to filter out specific protein release patterns.

Heterogeneity in study results may be greatly affected by differences in the COVID-19 case and control populations used in the five studies. For example, most studies focus on hospitalized patients, whereas the IMP and REP cohorts include both outpatients and inpatients suffering from end-stage kidney disease or going through hemodialysis. As another example, the MGH cohort’s control group consisted of ARDS patients who tested negative for COVID-19, whereas the control group for the IMP and REP cohorts consisted of COVID-19 negative ESKD patients undergoing hemodialysis. Similarly, the controls in both the GIM and ICU studies were COVID-19 negative patients with diseases such as cancer, diabetes, hypertension, chronic kidney disease, or heart diseases. Many of these significant differences must be considered when interpreting the findings because they may have an impact on the outcomes and comparability of the studies. Therefore, it is reasonable to assume that antecedent therapy and an imbalance in comorbidities between studies and their respective control groups could influence the circulating protein concentrations.

Comparative proteomics identified statistically robust overexpression of several inflammatory cytokines, specifically IL6, IL18, CCL7, CXCL10, and CXCL11, which could be targeted to prevent untoward immune-inflammatory effects in COVID-19 patients. Particularly, novel anti-human IL1R7 antibodies that block IL18 could be a promising COVID-19 treatment option. Thereby improving treatment options for the patients that display hypercytokinemia phenotypes, restoring the balance between pro- and anti-inflammatory cascades. Pertinently, validating differentially expressed proteins in five independent studies that may be significantly prognostic and can classify pathways that are amenable to current or future therapeutics.

## Materials and Methods

### Cohort ICU and GIM and COVID-19 Confirmation

#### Cohort Description

Sampling for the GIM study was conducted between March and April 2020 at New York-Presbyterian Hospital/Weill Cornell Medical Center under IRB# 19-10020914. GIM study is a single-center prospective study comparing hospitalized COVID-19 patients and non-COVID-19 control. The participants were adult (median age 64 years) of mixed race (Asian, Black, White, and other non-specific). All patients were screened for COVID-19 using SARS-CoV-2 RT-PCR. Hospitalized controls were selected based on negative RT-PCR results for SARS-CoV-2, and their samples were age/gender matched to COVID-19 patients. Children under the age of 18 year and pregnant women were excluded. Both COVID-19 patients and controls were suffering from heart diseases (14.5 vs. 26.0%; respectively), diabetes (25 vs. 18.0%), cancer (14.5 vs. 40.0%), hypertension (43.8 vs. 56.0%), and chronic kidney diseases (4.1 vs. 12.0%).

For ICU study, adult patients of mixed race (median age 53 years) were hospitalized at the New York-Presbyterian Hospital/Weill Cornell Medical Center between March and April 2020. The cohort is derived from the Weill Cornell Biobank of Critical Illness, a database that recruits and enrolls any patient admitted to Weill Cornell ICU. Demography of cohorts was recorded in the Weill Cornell Medicine COVID Institutional Data Repository (COVID-IDR), a high-quality manually abstracted registry of COVID-19 patients. Laboratories, ventilation parameters, vital signs and respiratory variables were obtained from the Weill Cornell-Critical Care Database for Advanced Research (WC-CEDAR). Missing or not available information was manually abstracted and registered into REDCap (40). The ICU patients were SARS-CoV-2 positive ARDS patients, and the controls were nonsepsis patients admitted to Presbyterian Hospital ICU. Patients’ recruitment, data collection, and sample processing procedures have previously been described (41, 42). In brief patients were excluded if they or their surrogate are unable to provide consent or if they were in a moribund state.

Clinical and laboratory data for the GIM and ICU studies were obtained from the Weill Cornell Medicine COVID Institutional Data Repository (COVID-IDR) and Weill Cornell-Critical Care Database for Advanced Research (WC-CEDAR). MGH dataset was downloaded from https://www.olink.com/mgh-covid-study/. The IMP and REP datasets were obtained from the supplementary data of https://www.medrxiv.org/content/10.1101/2020.11.05.20223289v1. GIM and ICU data were preprocessed using in-house scripts based on the maplet package (https://github.com/krumsieklab/maplet). All datasets were transferred into Summarized Experiment format (43).

### Plasma/Serum Proteomics

The Olink assays on ICU and GIM samples were performed using Inflammation (v.3021), Cardiovascular II (v.5005), and Cardiovascular III Panels (v.6113) (Olink, Uppsala, Sweden). Therefore, EDTA plasma and serum samples were heat-inactivated at 56°C for 15 minutes according to the virus inactivation protocol provided by Olink (www.olink.com/content/uploads/2020/04/CoronaVirus-Heat-Inactivation-webv1.pdf). The EDTA plasma or serum protein measurements were performed with the so-called Proximity Extension Assay technology (PEA) according to manufacturer’s instructions. In summary, high throughput real-time PCR of reporter DNA linked to protein specific antibodies was performed on a 96-well integrated fluidic circuits chip (Fluidigm, San Francisco, CA). Signal quantification was carried out on a Biomark HD system (Fluidigm, San Francisco, CA). Each sample was spiked with quality controls to monitor the incubation, extension, and detection steps of the assay. Additionally, samples representing external, negative, and inter-plate controls were included in each analysis run. From raw data, real time PCR cycle threshold (Ct) values were extracted using the Fluidigm RT-PCR analysis software at a quality threshold of 0.5 and linear baseline correction. Ct values were further processed using the Olink NPX manager software (Olink, Uppsala, Sweden). Here, log_2_-transformed Ct values from each sample and analyte were normalized based on spiked-in extension controls and scale-inverted to obtain Normalized log2 scaled Protein eXpression (NPX) values. NPX values were further adjusted based on the median of inter plate controls (IPC) for each protein and intensity median scaled between all samples and plates.

### Statistical Analysis

Statistical analyses were performed using R (version 4.0.4) (44). Linear models protein ~ state were computed using the R package MatrixEQTL (45), where ‘protein’ denotes scaled (mean = 0, s.d. = 1) Olink NPX values and state is coded 0=CovidNegative and 1=CovidPositive. Estimated effect sizes (beta) thus correspond to differences between cases and controls in relative units (s.d.), where positive betas correspond to higher protein levels observed in COVID-19 positive samples. Analyses were limited to proteins that were measured in all five studies. In cases where multiple time points were collected for a same patient, only the first data point was retained, which generally corresponds to time of admission. Summary statistics for all studies are provided as Supplementary Materials in PDF format generated using Rmarkdown. Summary statistics are further visualized as volcano plots by study and as scatterplots of effect sizes by pairwise study-to-study comparison. Finally, proteins that satisfy different levels of replication criteria are presented as heatmaps. Bonferroni correction was used to determine significance levels for inclusion, combined with various criteria regarding consistency in effect direction and number of studies with significant associations (see **Supplementary Data 1** for details). For discovery, a significance level of p < 0.05/5/Nprotein was used (incl. accounting for testing for association in five studies in parallel) and for replication p < 0.05/Ndiscovery was applied, with Nprotein corresponding to the number of analyzed proteins and Ndiscovery to the number of proteins taken forward to replication by meeting the discovery significance level.

### Voronoi Treemaps, 2D Cluster, and Pathway Assignment Analysis

ICU Voronoi treemaps of median protein RFIs were calculated based on KEGG Ortholog BRITE category, pathway, and protein levels (46).2D Cluster analysis of beta values was performed with Genedata Analyst (Basel, Switzerland, v.13.0.1) using 50% valid values and Euclidean for protein distance calculation (linkage: complete). At study level, Cosine was used for distance calculation (linkage: complete). Pathway assignment of significant altered proteins was performed using the g:Profiler using pathway annotation from human Reactome, WikiPathways and KEGG databases (47).

## Data Availability Statement

The datasets presented in this study can be found in online repositories. The names of the repository/repositories and accession number(s) can be found in the article/**Supplementary Material**.

## Ethics Statement

IRB approvals for the ICU study were reviewed and approved by NYP/WCMC with reference number 20-05022072 and 1405015116. The GIM study protocols were reviewed and approved by NYP/WCMC under IRB numbers 19-10020914 and 20-05022072. The patients/participants provided their written informed consent to participate in this study.

## Author Contributions

All authors contributed to the article and approved the submitted version.

## Funding

This work was supported by the Biomedical Research Program at Weill Cornell Medicine in Qatar, a program funded by the Qatar Foundation.

## Conflict of Interest

The authors declare that the research was conducted in the absence of any commercial or financial relationships that could be construed as a potential conflict of interest.

## Publisher’s Note

All claims expressed in this article are solely those of the authors and do not necessarily represent those of their affiliated organizations, or those of the publisher, the editors and the reviewers. Any product that may be evaluated in this article, or claim that may be made by its manufacturer, is not guaranteed or endorsed by the publisher.
