# Supplementary material for: Identification of Robust Protein Associations With COVID-19 Disease Based on Five Clinical Studies
Source: Front Immunol. 2022 Jan 25;12:781100. doi: 10.3389/fimmu.2021.781100 (PMC8821526; doi:10.3389/fimmu.2021.781100)
Supplement: Supplementary file 1 [file DataSheet_1.zip › SuppData.html]

Identification of Robust Protein Associations With COVID-19 Disease Based on Five Clinical Studies


# Identification of Robust Protein Associations With COVID-19 Disease Based on Five Clinical Studies

#### Supplementary Data to Suhre et al., Frontiers in Immunology, 2022

#### https://www.frontiersin.org/articles/10.3389/fimmu.2021.781100/full

## Material

### Data sources

- MGH dataset from https://www.olink.com/mgh-covid-study/
- Imperial datasets (IMP and REP) from supplementary data of https://www.medrxiv.org/content/10.1101/2020.11.05.20223289v1
- GIM and ICU data are here in the tabs “GIM data” and “ICU data”

### GIM data

### ICU data

## Results

### Overview

- a linear model PROT ~ COVID was used
- PROT are protein NPX values that are scaled to mean = 0 and s.d. = 1
- analysis limited to proteins available in all studies
- where times-series data was available, only the first data point per patient was used
- heatmaps: an association is replicated using all studies if p < 0.05/(5\*Nprotein) in one study and p < 0.05/(Ndiscovery) in one other
- scatterplots: an association is called replicated between two studies if p < 0.05/(2\*Nprotein) in one study and p < 0.05/(Ndiscovery) in the other

### Heatmap

```
## Proteins shown in below heatmap: CCL16 CCL7 CXCL10 CCL8 LGALS9 CXCL11 IL1RN CCL2 CD274 IL6 IL18 MERTK IFNG IL18R1
```

```
## Proteins shown in below heatmap: KITLG CDH5 BOC CCL16 PTX3 MERTK TNF ACP5 IL1RN CCL2 CD274 IL6 IL18 IFNG IL18R1 CCL8 LGALS9 CXCL11 GRN CCL7 CXCL10
```

```
## Proteins shown in below heatmap: CCL17 MMP12 BOC CCL16 KITLG CDH5 PRSS27 CNTN1 CD6 IFNG IL18R1 CCL8 LGALS9 CXCL11 GRN CCL7 CXCL10 PTX3 MERTK TNF ACP5 IL1RN CCL2 CD274 IL6 IL18 CXCL8 CDCP1 S100A12
```

```
## Proteins shown in below heatmap: TNFRSF10C CDH5 IL1R1 ALCAM ITGB2 BOC CCL16 CCL24 CCL17 MMP12 TNFSF12 KITLG PRSS27 CNTN1 CD6 IFNG IL18R1 CCL8 LGALS9 CXCL11 GRN CCL7 CXCL10 THBD FAS SELP IL17RA PCSK9 PTX3 TNFSF14 PRTN3 BLMH LIFR ADA CTSD CASP8 HMOX1 PARP1 IL33 IL10 IL4R TNFRSF1B CTSL IL1RL1 CCL3 CCL20 TNFRSF10B CDCP1 S100A12 CXCL8 GDF15 MB IL1RN CCL2 CD274 IL6 IL18 ACP5 MERTK TNF IL18BP SFTPD AGER
```

```
## Proteins shown in below heatmap: CCL16 TNFSF11 CCL7 CXCL10 CCL8 LGALS9 CXCL11 IL1RN CCL2 CD274 IL6 IL18 MERTK IFNG IL18R1
```

```
## Proteins shown in below heatmap: CCL17 MMP12 BOC CCL16 KITLG TNFSF11 PON3 F2R KLK6 EPCAM CD5 CDH5 PRSS27 CNTN1 CD6 IL1RN CCL2 CD274 IL6 IL18 IFNG IL18R1 CCL8 LGALS9 CXCL11 GRN CCL7 CXCL10 TNFSF13B MERTK PLAT CSF1 TNF CXCL1 IL7 PTX3 SIRT2 CXCL8 S100A12 VEGFA CDCP1 CPA1 ACP5 CPB1 TNFRSF10A
```

```
## Proteins shown in below heatmap: EPCAM CD5 LGALS4 F2R KLK6 FABP2 AMBP CCL11 RARRES2 CCL25 SELPLG NPPB DLK1 CCL17 MMP12 BOC CCL16 KITLG TNFSF11 PON3 CDH5 PRSS27 CNTN1 CD6 IL1RN CCL2 CD274 IL6 IL18 IFNG IL18R1 CCL8 LGALS9 CXCL11 GRN CCL7 CXCL10 IL12B IL22RA1 PDGFB SPP1 SLAMF1 HAO1 CXCL8 S100A12 VEGFA CDCP1 CX3CL1 CTSZ HBEGF CA5A STK4 CPA1 IL6R ACP5 CPB1 TNFRSF10A SIRT2 TNFSF13B SORT1 DECR1 MERTK PLAT CSF1 TNF CXCL1 IL7 PTX3
```

### Volcano plots

### Scatterplots

### All

### MGH

### IMP

### REP

### GIM

### ICU

## References

- Rmarkdown-cookbook
- DT::datatable
- Rmarkdown homepage
- flexdashboard
- shiny
